# Impact of marital status on diagnosis and survival of patients with ocular cancer: An observational study

**DOI:** 10.1097/MD.0000000000044162

**Published:** 2025-08-29

**Authors:** Yu Zhou, Yi Feng, Min Zhang

**Affiliations:** a Department of Ophthalmology, The Affiliated Chengdu 363 Hospital of Southwest Medical University, Chengdu, China; b Department of Electrocardiogram, The Affiliated Chengdu 363 Hospital of Southwest Medical University, Chengdu, China.

**Keywords:** diagnosis, marital status, ocular cancer, population characteristics, survival

## Abstract

Marital status may influence cancer management by affecting factors such as emotional support, health-related decision-making, and access to medical care. Its impact has been reported across various cancer types. However, the role of marital status in the diagnosis and survival of ocular cancer remains underexplored. Patients diagnosed with ocular cancer between 2000 and 2019 were identified from the Surveillance, Epidemiology, and End Results database, a population-based cancer registry in the United States. Multivariable ordinal logistic regression was used to evaluate the association between marital status and stage at diagnosis. Propensity score matching was used to match baseline characteristics between married and unmarried patients, and overall survival (OS) and cancer-specific survival (CSS) were estimated using Kaplan–Meier curves. A total of 7556 patients were included. Unmarried patients were more likely to be present with advanced disease at diagnosis than married patients (OR = 1.31, 95% CI, 1.11–1.55; *P* = .001). After propensity score matching, 2625 married and 2625 unmarried patients were included. Unmarried patients showed significantly worse OS (HR = 1.40, 95% CI, 1.29–1.52, *P* < .001) and CSS (HR = 1.13, 95% CI, 1.02–1.26, *P* = .022) compared with married patients. Subgroup analysis suggests that females (female vs male; OS: HR 1.45 vs 1.33; CSS: HR 1.18 vs 1.07), older individuals (>60 vs 18–60; OS: HR 1.51 vs 1.17; CSS: HR 1.36 vs 1.23), and low-risk populations (localized [localized vs regional vs distant; OS: HR 1.47 vs 1.20 vs 1.30; CSS: HR 1.21 vs 1.15 vs 1.18], low grade [Grade I–II vs Grade III–IV; OS: HR 1.87 vs 1.14; CSS: HR 1.70 vs 1.08], nonmelanoma [nonmelanoma vs melanoma; OS: HR 1.50 vs 1.34; CSS: HR 1.22 vs 1.11]) appear to derive greater survival benefits from marriage. Additionally, the survival disparity attributable to marriage has increased over time (2010–2019 vs 2000–2009: OS, HR 1.45 vs 1.36; CSS, HR 1.22 vs 1.11). In the multivariable Cox regression analysis, unmarried status was identified as an independent predictor of poor OS (HR = 1.37, 95% CI, 1.27–1.49, *P* < .001) and CSS (HR = 1.12, 95% CI, 1.02–1.22, *P* = .031). In ocular cancer patients, unmarried individuals are typically diagnosed at later disease stages and have poorer survival outcomes.

## 1. Introduction

Ocular and orbital cancers are rare malignancies worldwide. It is estimated that there are approximately 6679 to 7095 new cases of uveal melanoma and 7202 to 8102 new cases of retinoblastoma annually across the globe.^[1,2]^ In the United States, approximately 3000 new cases are diagnosed each year and approximately 500 deaths are attributed to these cancers.^[3–5]^ Although the incidence of ocular cancer is relatively low, it significantly impacts the quality of life and survival prognosis of patients.^[6,7]^ Ocular cancer encompasses a variety of histological types, with melanoma being the most common in adult patients.^[3]^Among adult patients, approximately 83% of ocular melanomas occur in the uvea, 5% in the conjunctiva, and the remaining 10% in other parts of the eye.^[8,9]^

Marital status plays an important role in the management of various cancers. Studies have shown that married patients have advantages over unmarried patients in several areas, including earlier cancer diagnosis, higher treatment compliance, and better prognosis.^[10]^ This phenomenon may be attributable to the greater psychological and financial support, as well as more regular lifestyles, that married patients often experience.^[11–13]^ In common cancers such as lung, breast, prostate, rectal, cervical, and kidney cancers, the protective effect of marriage has been extensively studied.^[14–19]^ However, the impact of marital status on ocular cancer patients remains insufficiently explored.^[20–22]^ Two studies have examined the relationship between marital status and prognosis in patients with uveal melanoma,^[20,21]^ and one study has investigated this association in patients with ocular and periocular malignancies.^[22]^ However, these studies usually have limitations, such as being limited to a single histology, lacking the impact of marital status on diagnosis and the impact on survival of patients with different characteristics.

Based on the Surveillance, Epidemiology, and End Results (SEER) database, this study aimed to comprehensively explore the impact of marital status in adult patients with ocular cancer (excluding orbital cancers), especially its role in cancer diagnosis and prognosis of patients with different characteristics.

## 2. Patients and methods

### 2.1. Data source

Ocular cancers are rare malignancies, and large-scale population-based data on their epidemiology and prognosis are limited worldwide. This rarity presents challenges for conducting comprehensive and reliable analyses. The SEER database, established by the National Cancer Institute, provides high-quality data on cancer incidence, treatment, and survival, covering approximately half population of the United States.^[23,24]^

The inclusion of individual cases in SEER depends on mandatory reporting from hospitals, pathology laboratories, and other healthcare facilities within participating regions. In accordance with cancer reporting regulations, medical institutions are required to report newly diagnosed cancer cases to their regional cancer registries. Certified tumor registrars abstract detailed patient information from medical records, pathology reports, and treatment summaries, including demographics, tumor site, histology, staging at diagnosis, and treatment modalities. These data are coded using standardized protocols and undergo rigorous quality control procedures before being incorporated into the SEER database.^[25]^

Due to the large volume of data in the SEER database, it provides significant support for epidemiological and clinical research on cancer, particularly rare cancers. In this study, we utilized the SEER-17 dataset, updated in April 2024.^[26]^ This study was based on publicly available, de-identified data from the SEER database; therefore, ethical approval was waived, and informed consent was not required.

### 2.2. Patient selection

Primary ocular cancers (excluding orbital cancer, C69.0–C69.5, C69.8–C69.9) were identified based on the International Classification of Diseases for Oncology, 3rd edition codes. Adult patients (aged ≥ 18 years) diagnosed with ocular cancer between 2000 and 2019 were included in this study. We excluded patients under the age of 18 because the SEER database is based on the U.S. population, and marital status (our key variable of interest) is generally not applicable to individuals under 18, as the legal age for marriage in most U.S. states is typically 18 years. Patients with a diagnosis recorded only by autopsy or death certificate, multiple malignancies, or lack of data on specific demographic variables, including race, marital status, median household income (adjusted to 2022), region of residence, or tumor outcome were excluded. The excluded patients represented <5% of the overall population.

### 2.3. Statistical analysis

Due to the small sample size caused by the rarity of ocular cancer, continuous and categorical variables were simplified to improve the feasibility and interpretability of the statistical analysis. The diagnosis years were grouped into 2000 to 2009 and 2010 to 2019 to examine whether the survival benefit associated with marital status changed over time in patients with ocular cancer. Age was divided based on the median age of the included patients into 2 groups: 18 to 60 and >60. Median household income was categorized as <80,000 United States Dollar and >80,000 United States Dollar. Race was classified as White, Black, or Other (including Asian or Pacific Islander and American Indian/Alaska Native). Given the histological diversity and relative rarity of ocular malignancies other than melanoma in adults, these were collectively categorized as nonmelanoma. Consequently, the histological classification was divided into melanoma and nonmelanoma. In accordance with previous studies, marital status at diagnosis was simplified into 2 categories: married and unmarried, with the latter including single, separated, divorced, widowed, and unmarried or domestic partner.^[10]^

The primary focus of this study was to assess the impact of marital status on diagnosis and prognosis in adult patients with ocular cancer. Differences in baseline characteristics between married and unmarried groups were tested using Pearson Chi-squared test. To evaluate the impact of marital status on cancer staging at diagnosis, a multivariable ordinal logistic regression model was used, with cancer stage as the ordered dependent variable (localized < regional < distant) and marital status as the primary independent variable. The model was adjusted for diagnosis year, age, sex, race, residence, median household income, tumor grade, and histology. Subsequently, Kaplan–Meier curves were used to assess overall survival (OS) and cancer-specific survival (CSS) for married and unmarried patients, with log-rank tests comparing the survival differences between the 2 groups. In order to control the possible bias caused by differences in baseline characteristics, a 1:1 propensity score matching (PSM) method was used to match married and unmarried patients. After matching, Kaplan–Meier curves were reestimated for both groups’ OS and CSS, and log-rank tests were used to further evaluate survival differences. Considering previous studies suggest that the protective effect of marriage on survival may differ by sex, subgroup analyses based on sex were performed.^[10,27]^ In addition, to comprehensively evaluate the impact of marital status on the prognosis of patients with different characteristics of ocular cancer, we also performed subgroup analyses based on all predetermined variables (including year of diagnosis, age, sex, race, place of residence, median household income, stage, grade, and histological type). Finally, multivariable Cox regression analysis was conducted to evaluate the independent effect of marital status on OS and CSS. All statistical analyses were performed using R software (Version 4.3.0; R Foundation for Statistical Computing, Vienna, Austria), and a 2-sided *P* value < .05 was considered statistically significant.

## 3. Results

### 3.1. Patient baseline characteristics

As shown in Table 1, a total of 7556 patients were included in this study, of which 4847 (64.1%) were married and 2709 (35.9%) were unmarried. Compared with married patients, unmarried patients were older (>60 years: 55.3% vs 49.5%, *P* < .001), had a higher proportion of females (57.1% vs 40.3%, *P* < .001), a higher proportion of Black patients (2.6% vs 1.4%, *P* < .001), and were more likely to reside in urban areas (87.3% vs 85.4%, *P* = .02). In addition, unmarried patients were more likely to have nonmelanoma histology (36.0% vs 31.6%). Moreover, significant differences were also observed between the 2 groups in terms of disease stage (*P* = .003), local surgery (*P* < .001), and radiotherapy (*P* < .001). However, marital status was not significantly associated with year of diagnosis, median household income, grade, or chemotherapy (all *P* > .05). After PSM, 2625 married and 2625 unmarried patients were included, and the baseline characteristics were well balanced between the 2 groups (all *P* > .05).

**Table 1:** Patient baseline characteristics before and after propensity score matching.

Characteristics	Unmatched			Matched		
	Married, N = 4847	Unmarried, N = 2709	*P*-value	Married, N = 2625	Unmarried, N = 2625	*P*-value
Year of diagnosis			.568			.558
2000–2009	2146 (44.3%)	1181 (43.6%)		1120 (42.7%)	1141 (43.5%)	
2010–2019	2701 (55.7%)	1528 (56.4%)		1505 (57.3%)	1484 (56.5%)	
Age, year			<.001			.641
18–60	2446 (50.5%)	1212 (44.7%)		1218 (46.4%)	1201 (45.8%)	
˃60	2401 (49.5%)	1497 (55.3%)		1407 (53.6%)	1424 (54.2%)	
Sex			<.001			.846
Male	2892 (59.7%)	1162 (42.9%)		1169 (44.5%)	1162 (44.3%)	
Female	1955 (40.3%)	1547 (57.1%)		1456 (55.5%)	1463 (55.7%)	
Race			<.001			.687
White	4640 (95.7%)	2569 (94.8%)		2511 (95.7%)	2498 (95.2%)	
Black	66 (1.4%)	71 (2.6%)		53 (2.0%)	58 (2.2%)	
Others	141 (2.9%)	69 (2.5%)		61 (2.3%)	69 (2.6%)	
Residence			.020			.805
Urban	4140 (85.4%)	2366 (87.3%)		2289 (87.2%)	2283 (87.0%)	
Rural	707 (14.6%)	343 (12.7%)		336 (12.8%)	342 (13.0%)	
Median household income			.104			.502
˂80,000 USD	2710 (55.9%)	1567 (57.8%)		1521 (57.9%)	1545 (58.9%)	
˃80,000 USD	2137 (44.1%)	1142 (42.2%)		1104 (42.1%)	1080 (41.1%)	
Stage			.003			.564
Localized	3213 (66.3%)	1711 (63.2%)		1697 (64.6%)	1672 (63.7%)	
Regional	302 (6.2%)	203 (7.5%)		190 (7.2%)	200 (7.6%)	
Distant	123 (2.5%)	98 (3.6%)		72 (2.7%)	88 (3.4%)	
Unknown/unstaged	1209 (24.9%)	697 (25.7%)		666 (25.4%)	665 (25.3%)	
Grade			.225			.638
Grade I–II	298 (6.1%)	189 (7.0%)		164 (6.2%)	181 (6.9%)	
Grade III–IV	149 (3.1%)	94 (3.5%)		92 (3.5%)	91 (3.5%)	
Unknown	4400 (90.8%)	2426 (89.6%)		2369 (90.2%)	2353 (89.6%)	
Histology			<.001			>.999
Melanoma	3315 (68.4%)	1733 (64.0%)		1693 (64.5%)	1693 (64.5%)	
Non-melanoma	1532 (31.6%)	976 (36.0%)		932 (35.5%)	932 (35.5%)	
Local surgery			<.001			.375
Yes	1900 (39.2%)	1258 (46.4%)		1215 (46.3%)	1183 (45.1%)	
No/unknown	2947 (60.8%)	1451 (53.6%)		1410 (53.7%)	1442 (54.9%)	
Radiotherapy			<.001			.760
Yes	3074 (63.4%)	1478 (54.6%)		1467 (55.9%)	1478 (56.3%)	
No/unknown	1773 (36.6%)	1231 (45.4%)		1158 (44.1%)	1147 (43.7%)	
Chemotherapy			.232			.157
Yes	235 (4.8%)	115 (4.2%)		94 (3.6%)	114 (4.3%)	
No/unknown	4612 (95.2%)	2594 (95.8%)		2531 (96.4%)	2511 (95.7%)	

USD = United States Dollar.

### 3.2. Marriage and stage at diagnosis

As shown in Table 2, after adjusting for covariates, unmarried patients were more likely to present with advanced disease at diagnosis compared to married patients (OR = 1.31, 95% CI, 1.11–1.55, *P* = .001). Other factors associated with advanced disease included older age (OR = 1.22, 95% CI, 1.04–1.44, *P* = .017), minority race (White as the reference group; Black: OR = 1.84, 95% CI, 1.17–2.83, *P* = .007; Others: OR = 2.16, 95% CI, 1.49–3.07, *P* < .001), higher grade (OR = 4.78, 95% CI, 3.30–6.96, *P* < .001), and nonmelanoma histology (OR = 2.70, 95% CI, 2.26–3.22, *P* < .001). However, no significant differences were observed between residence (*P* = .388), median household income (*P* = .067), sex (*P* = .323), and diagnosis year (*P* = .775) in relation to cancer stage distribution.

**Table 2:** Ordered logistic regression identifies factors affecting staging of patients with ocular cancer.

Characteristic	Univariable		Multivariable	
	OR (95% CI)	*P*-value	OR	*P*-value
Year of diagnosis				
2000–2009	Ref		Ref	
2010–2019	0.98 (0.84–1.16)	.848	0.98 (0.82–1.16)	.775
Age				
18–60	Ref		Ref	
˃60	1.26 (1.08–1.48)	.004	1.22 (1.04–1.44)	.017
Sex				
Male	Ref		Ref	
Female	0.87 (0.74–1.01)	.073	0.92 (0.78–1.09)	.323
Race				
White	Ref		Ref	
Black	3.03 (1.97–4.53)	<.001	1.84 (1.17–2.83)	.007
Others	2.70 (1.90–3.77)	<.001	2.16 (1.49–3.07)	<.001
Marital status				
Married	Ref		Ref	
Unmarried	1.34 (1.14–1.56)	<.001	1.31 (1.11–1.55)	.001
Median household income				
˂80,000 USD	Ref		Ref	
˃80,000 USD	0.84 (0.72–0.99)	.034	0.85 (0.71–1.01)	.067
Residence				
Urban	Ref		Ref	
Rural	0.92 (0.73–1.15)	.466	0.90 (0.70–1.14)	.388
Grade				
Grade I–II	Ref		Ref	
Grade III–IV	4.58 (3.18–6.65)	<.001	4.78 (3.30–6.96)	<.001
Unknown	0.58 (0.44–0.76)	<.001	1.14 (0.85–1.53)	.391
Histology				
Melanoma	Ref		Ref	
Non-melanoma	3.36 (2.86–3.94)	<.001	2.70 (2.26–3.22)	<.001

USD = United States Dollar.

### 3.3. Marriage and survival

**Figure 1.**
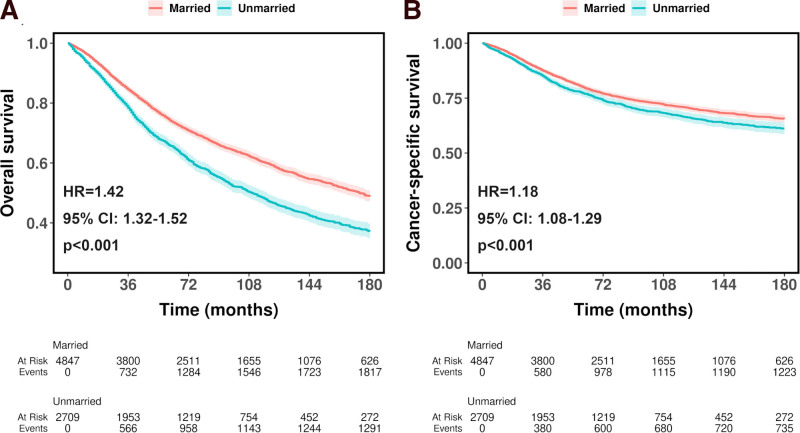
Kaplan–Meier curves for overall survival (A) and cancer-specific survival (B) of patients diagnosed with ocular cancer before propensity score matching.

**Figure 2.**
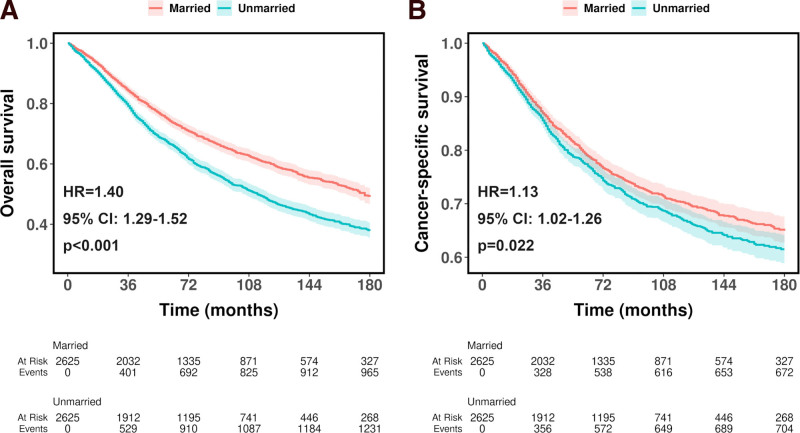
Kaplan–Meier curves for overall survival (A) and cancer-specific survival (B) of patients diagnosed with ocular cancer after propensity score matching.

As shown in Fig. 1, before PSM, unmarried patients showed worse OS (HR = 1.42, 95% CI, 1.32–1.52, *P* < .001, Fig. 1A) and CSS (HR = 1.18, 95% CI, 1.08–1.29, *P* < .001, Fig. 1B) compared to married patients. After PSM, unmarried patients showed a reduced but still significantly worse OS (HR = 1.40, 95% CI, 1.29–1.52, *P* < .001, Fig. 2A) and CSS (HR = 1.13, 95% CI, 1.02–1.26, *P* = .022, Fig. 2B) than married patients. Table S1, Supplemental Digital Content, https://links.lww.com/MD/P794 shows the OS and CSS rates at 36, 60, 120, and 180 months before and after PSM. Compared with married patients, unmarried patients showed worse OS and CSS at all stages.

**Figure 3.**
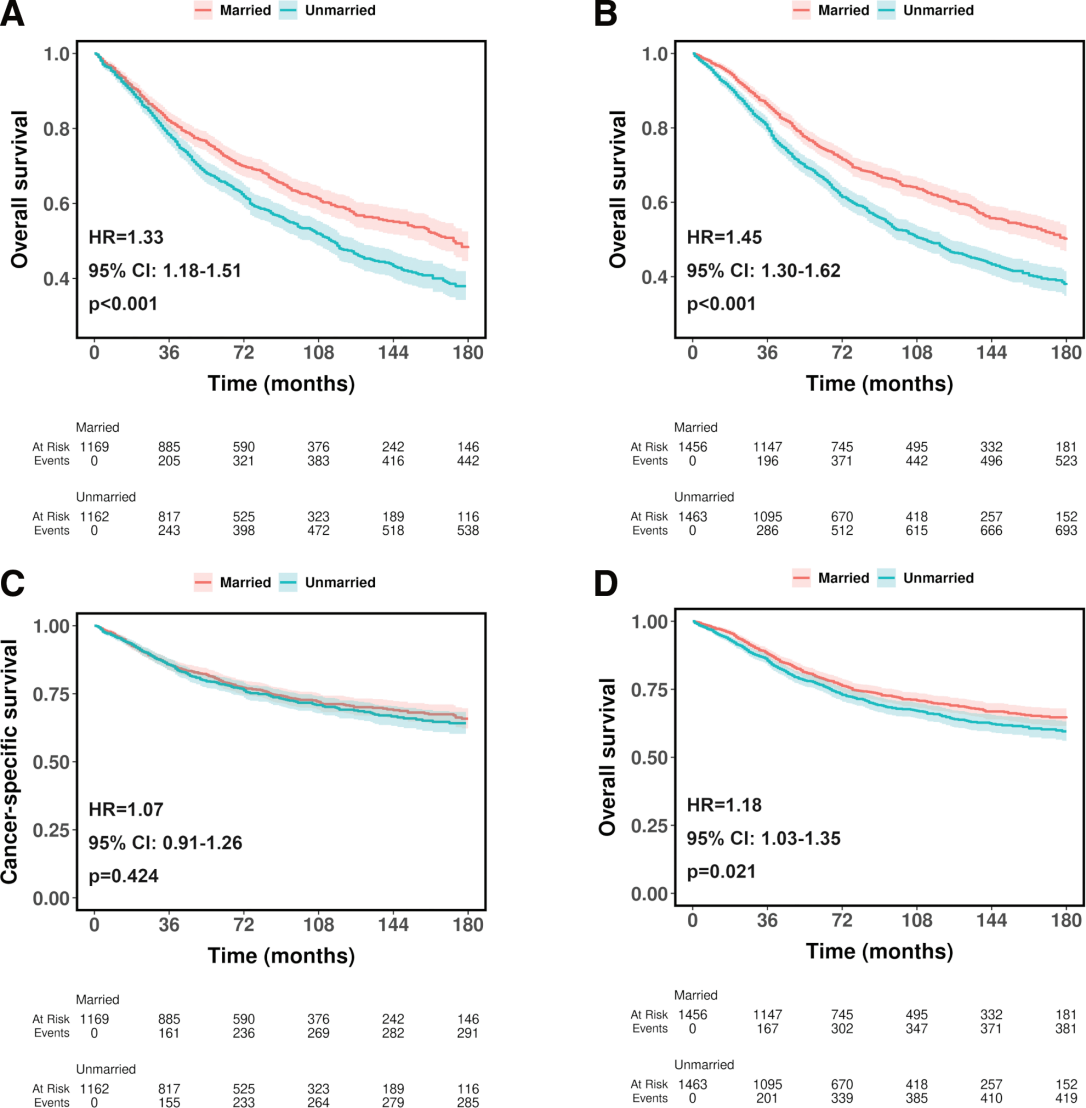
Subgroup analysis based on sex of overall survival and cancer-specific survival in patients diagnosed with ocular cancer after propensity score matching. (A) Overall survival in male patients. (B) Overall survival in female patients. (C) Cancer-specific survival in male patients. (D) Cancer-specific survival in female patients.

**Figure 4.**
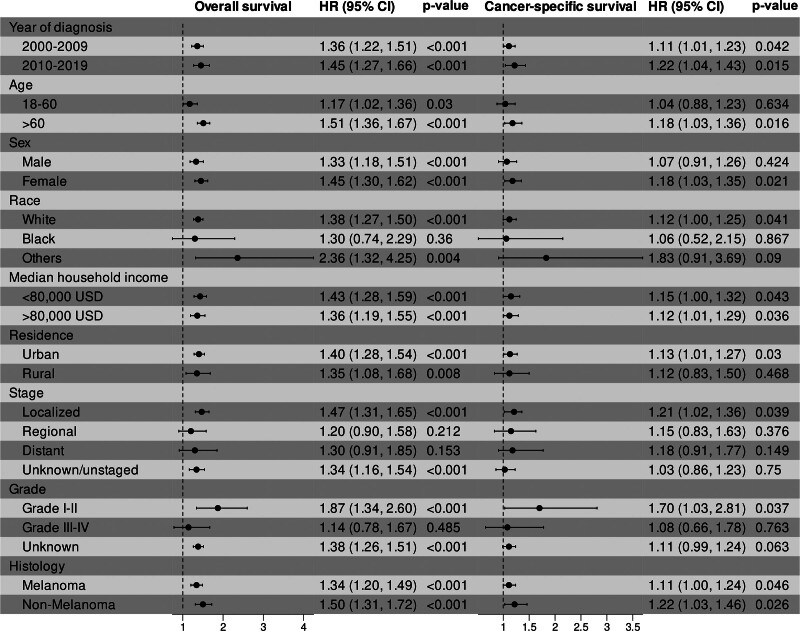
Subgroup analyses of overall survival and cancer-specific survival based on all prespecified variables after propensity score matching.

Subgroup analysis based on sex is shown in Fig. 3. In terms of OS, both unmarried male patients (HR = 1.33, 95% CI, 1.18–1.51, *P* < .001, Fig. 3A) and unmarried female patients (HR = 1.45, 95% CI, 1.30–1.62, *P* < .001, Fig. 3B) showed worse OS compared with married patients. In terms of CSS, unmarried male patients did not show significant differences compared with married patients (HR = 1.07, 95% CI, 0.91–1.26, *P* = .424, Fig. 3C), while unmarried female patients showed worse CSS (HR = 1.18, 95% CI, 1.03–1.35, *P* = .021, Fig. 3D). Figure 4 displays the results of all subgroup analyses based on prespecified variables. For OS, unmarried patients exhibited worse survival in all subgroups (all HR > 1), although the differences did not reach statistical significance in Black patients (*P* = .36), those with regional (*P* = .212) or distant (*P* = .153) stage, and high-grade patients (*P* = .485). For CSS, unmarried patients also had worse survival in all subgroups (all HR > 1), but no significant differences were observed in several subgroups, including those aged 18 to 60 years (*P* = .634), Black (*P* = .867) and Other races (*P* = .09), rural residence (*P* = .468), regional (*P* = .376), distant (*P* = .149), and unknown/unstaged (*P* = .75) stages, high grade (*P* = .763), and unknown grade (*P* = .063).

Table 3 presents the results of the multivariable Cox regression analysis. After adjusting for covariates, unmarried patients still showed significantly worse OS (HR = 1.37, 95% CI, 1.27–1.49, *P* < .001) and CSS (HR = 1.12, 95% CI, 1.02–1.22, *P* = .031) compared to married patients.

**Table 3:** Multivariate Cox regression of overall and cancer-specific survival in patients with ocular cancer.

Characteristic	Overall survival		Cancer-specific survival	
	HR (95% CI)	*P*-value	HR (95% CI)	*P*-value
Year of diagnosis				
2000–2009	Ref		Ref	
2010–2019	0.88 (0.80–0.97)	.01	0.92 (0.82–1.04)	.186
Age, year				
18–60	Ref		Ref	
˃60	2.76 (2.52–3.02)	<.001	1.74 (1.56–1.94)	<.001
Sex				
Male	Ref		Ref	
Female	0.85 (0.78–0.92)	<.001	1.01 (0.91–1.13)	.839
Race				
White	Ref		Ref	
Black	1.13 (0.86–1.50)	.381	1.11 (0.77–1.57)	.617
Others	0.87 (0.66–1.15)	.341	0.86 (0.61–1.21)	.385
Marital status				
Married	Ref		Ref	
Unmarried	1.37 (1.27–1.49)	<.001	1.12 (1.02–1.22)	.031
Median household income				
˂80,000 USD	Ref		Ref	
˃80,000 USD	0.94 (0.86–1.03)	.198	0.97 (0.86–1.08)	.56
Residence				
Urban	Ref		Ref	
Rural	1.08 (0.95–1.22)	.237	1 (0.84–1.17)	.96
Stage				
Localized	Ref		Ref	
Regional	1.57 (1.34–1.83)	<.001	1.99 (1.64–2.40)	<.001
Distant	5.32 (4.38–6.45)	<.001	8.24 (6.68–10.16)	<.001
Unknown/unstaged	1.33 (1.20–1.47)	<.001	1.56 (1.37–1.77)	<.001
Grade				
Grade I–II	Ref		Ref	
Grade III–IV	1.31 (1.02–1.65)	.033	1.45 (1.01–2.07)	.042
Unknown	1.2 (1.00–1.43)	.045	1.64 (1.26–2.13)	<.001
Histology				
Melanoma	Ref		Ref	
Non-melanoma	0.87 (0.78–0.96)	.007	0.76 (0.67–0.87)	<.001
Local surgery				
Yes	Ref		Ref	
None-unknown	0.87 (0.77–0.97)	.014	0.74 (0.64–0.85)	<.001
Radiotherapy				
Yes	Ref		Ref	
None/unknown	1.42 (1.27–1.58)	<.001	1.26 (1.10–1.44)	<.001
Chemotherapy				
Yes	Ref		Ref	
No/unknown	0.78 (0.64–0.95)	.011	0.73 (0.58–0.91)	.006

USD = United States Dollar.

## 4. Discussion

The protective effects of marriage have been extensively explored for a variety of diseases, both benign and malignant.^[10,28–30]^ In the field of cancer, one of the most notable studies is that of Aizer et al., which found that among the top ten causes of cancer-related death in the United States, married patients were less likely to present with metastatic disease (OR = 0.83, 95% CI, 0.82–0.84, *P* < .001), more likely to receive definitive treatment (OR = 1.53, 95% CI, 1.51–1.56, *P* < .001), and had a lower risk of death from cancer (HR = 0.80, 95% CI, 0.79–0.81, *P* < .001).^[10]^ This trend remained significant when analyzing each cancer separately. Surprisingly, for 5 cancers (prostate, breast, colorectal, esophageal, and head & neck) the survival benefit of marriage was even greater than the effect of chemotherapy.^[10]^ Several mechanisms have been proposed to explain this association. Married individuals may receive more emotional support, encouragement to seek timely medical care, and assistance with treatment adherence, all of which can contribute to better outcomes. These psychosocial benefits of marriage are likely to be consistent across different sociocultural and healthcare settings.^[10–12]^ In the United States specifically, health insurance coverage is often linked to employment or family status, and being married may facilitate better access to medical services. Therefore, although the positive impact of marriage on survival has been observed in various contexts, certain aspects of this association, particularly those related to healthcare access, may be especially relevant within the U.S. healthcare system.^[31]^

Nevertheless, although the role of marriage has been extensively investigated in common cancers, detailed research on its role in rare cancers remains limited. In the field of ocular cancer, Alfaar et al and Cai et al found that married patients showed better survival in uveal melanoma, and Loya et al confirmed that marriage was associated with better survival in ocular and periocular malignancies.^[20–22]^ However, Alfaar et al and Cai et al only focused on uveal melanoma and lacked attention to other types of ocular cancer.^[20,21]^ The study of Loya et al only included adults over 25 years old, which may limit the generalizability of the results.^[22]^ In addition, their analysis failed to explore the relationship between marriage and diagnosis stage, nor did it clarify the impact of marriage on different populations. Previous studies have shown that the impact of marriage on different populations may be different.^[10,27]^ Therefore, further research on the role of marriage in patients with ocular cancer, especially considering the characteristics of different populations, is still particularly important.

In this study, we identified that being unmarried is associated with a higher risk of advanced disease and worse survival (including OS and CSS) in ocular cancer. This suggests that improving early diagnosis, especially in unmarried patients, may help improve their prognosis. In the subgroup analysis, we observed consistent trends across all subgroups for both OS and CSS (all HR > 1); however, the results were not statistically significant in several subgroups, including patients aged 18 to 60 years (CSS), males (CSS), Black (OS and CSS) and Other races (CSS), those residing in rural areas (CSS), patients with regional (OS and CSS), distant (OS and CSS), or unknown/un-staged disease (CSS), and those with Grade III to IV (OS and CSS) or unknown tumor grade (CSS). Due to the rarity of ocular cancer, the limited sample sizes may have reduced the statistical power to detect significant associations. Therefore, we focused more on the comparison of the degree of benefit from marriage in patients with different characteristics. Sex differences were observed in the survival disadvantage caused by being unmarried, whether in terms of OS (female vs male; HR 1.45 vs 1.33) or CSS (female vs male; HR 1.18 vs 1.07). Female ocular cancer patients seem to gain more survival benefits from marriage, which was consistent with other cancers.^[10,27]^ Age was also an important factor affecting the survival benefit brought by marriage. Elderly ocular cancer patients benefited more from marriage than younger patients (>60 vs 18–60; OS: HR 1.51 vs 1.17; CSS: HR 1.36 vs 1.23). Another interesting finding is that the low-risk population appears to derive more survival benefit from marriage. Patients with localized stage (localized vs regional vs distant; OS: HR 1.47 vs 1.20 vs 1.30; CSS: HR 1.21 vs 1.15 vs 1.18), low grade (Grade I–II vs Grade III–IV; OS: HR 1.87 vs 1.14; CSS: HR 1.70 vs 1.08), and nonmelanoma (nonmelanoma vs melanoma; OS: HR 1.50 vs 1.34; CSS: HR 1.22 vs 1.11) cancers experience greater survival benefits from marriage, and these factors were identified as independent prognostic factors for better outcomes in the Cox regression analysis (Table 3). Additionally, the survival difference attributable to marriage has increased over time (2010–2019 vs 2000–2009; OS, HR 1.45 vs 1.36; CSS, HR 1.22 vs 1.11). This may be due to advancements in treatment and diagnostic techniques in recent years, which allow married patients to detect diseases earlier and receive more effective treatments, thereby enhancing the survival benefits of marriage.^[32,33]^ In summary, these findings suggest that there are differences in survival benefits derived from marriage across various populations, highlighting the complex and multifaceted impact of marital status on survival, which necessitates personalized interventions based on patients’ characteristics.

## 5. Limitations

This study provides a comprehensive analysis of the role of marriage in ocular cancer patients, particularly considering the differences across populations with varying characteristics. However, there are several limitations to note. First, due to the complexity of treatment modalities for ocular cancer, the SEER database lacks detailed information on therapeutic regimens such as chemotherapy agents, radiotherapy protocols, and treatment adherence. This prevents an in-depth evaluation of how marital status may influence specific treatment decisions. Second, while the SEER database includes a large and diverse population, it does not cover the entire United States, raising the possibility of selection bias. Moreover, SEER is specific to the U.S., and no comparable population-based database with sufficient ocular cancer cases exists in other countries, limiting the generalizability of our findings beyond the U.S. population. Third, marital status is only recorded at the time of diagnosis and may change throughout the course of disease; however, such changes cannot be captured in the dataset, potentially introducing misclassification bias. Lastly, the classification of all non-married individuals, including divorced, separated, widowed, and those in domestic partnerships, under the broad category of “unmarried” may obscure important differences among these subgroups.

## 6. Conclusion

In patients with ocular cancer, unmarried individuals are typically diagnosed at later disease stages and experience poorer survival outcomes. The survival disparity associated with marital status is influenced by factors such as gender, age, tumor stage, grade, and histological type. Moreover, the survival benefit attributed to marriage has increased in more recent diagnosis years. Future research should aim to explore the underlying mechanisms driving these disparities, including psychosocial support, treatment adherence, and access to care. Additionally, studies using prospective or international datasets could help validate and extend these findings to broader populations.

## Acknowledgments

We would like to express our gratitude to the SEER program, which provided the comprehensive data used in this study.

## Author contributions

**Conceptualization:** Yu Zhou, Yi Feng, Min Zhang.

**Data curation:** Yu Zhou, Yi Feng.

**Formal analysis:** Yu Zhou.

**Investigation:** Yu Zhou.

**Methodology:** Yu Zhou.

**Supervision:** Min Zhang

**Writing – original draft:** Yu Zhou, Min Zhang.

**Writing – review & editing:** Yu Zhou, Min Zhang.

## Correction

This article was originally published with an incorrect degree listed for Yu Zhou, Yi Feng, and Min Zhang. All of the authors’ degrees have been corrected from “MD” to “MMed” in the online version.

## Supplementary Material


