# Supplementary material for: Impact of marital status on diagnosis and survival of patients with ocular cancer: An observational study
Source: Medicine (Baltimore). 2025 Aug 29;104(35):e44162. doi: 10.1097/MD.0000000000044162 (PMC12401195; doi:10.1097/MD.0000000000044162)
Supplement: Supplementary file 1 [file medi-104-e44162-s001.docx]

**Table S1. Overall and cancer-specific survival rates by marriage in ocular cancer patients before and after propensity score matching**

| Residence | Survival rate (95% CI, %) | | | | p-value^*^ |
| --- | --- | --- | --- | --- | --- |
|  | 36-months | 60-months | 120-months | 180-months |  |
| OS after PSM |  |  |  |  |  |
| Married | 84.5 (83.5-85.5) | 74.6 (73.3-75.9) | 59.7 (58.1-61.3) | 48.8 (46.9-50.7) | <0.001 |
| Unmarried | 78.4 (76.8-80.0) | 66.0 (64.2-67.9) | 47.1 (44.9-49.4) | 37.1 (34.8-39.7) |  |
| CSS after PSM |  |  |  |  |  |
| Married | 87.5 (86.5-88.4) | 80.0 (78.8-81.2) | 70.9 (69.4-72.4) | 65.6 (63.8-67.4) | <0.001 |
| Unmarried | 85.0 (83.7-86.5) | 77.2 (75.5-78.9) | 66.4 (64.3-68.6) | 61.2 (58.6-63.8) |  |
| OS after PSM |  |  |  |  |  |
| Married | 84.3 (82.9-85.7) | 74.6 (72.9-76.4) | 60.3 (58.2-62.5) | 49.1 (46.5-51.8) | <0.001 |
| Unmarried | 79.2 (77.6-80.8) | 66.8 (64.9-68.7) | 47.8 (45.6-50.1) | 37.9 (35.4-40.5) |  |
| CSS after PSM |  |  |  |  |  |
| Married | 86.9 (85.6-88.3) | 79.7 (78.1-81.4) | 70.3 (68.2-72.3) | 65.0 (62.5-67.5) | 0.022 |
| Unmarried | 85.6 (84.2-87.0) | 77.7 (76.0-79.4) | 66.8 (64.7-69.1) | 61.5 (58.9-64.2) |  |

Abbreviation: CSS, cancer-specific survival; PSM, propensity score matching; OS, overall survival

**^*^**Log-rank test
